# Narratives of the Russian-Ukrainian conflict in the Italian press and social media

**DOI:** 10.3389/fsoc.2025.1651055

**Published:** 2025-10-31

**Authors:** Simona Gozzo, Stefania Fragapane

**Affiliations:** ^1^Department of Political and Social Sciences, University of Catania, Catania, Italy; ^2^Faculty of Social Sciences and Communication, Universitas Mercatorum, Rome, Italy

**Keywords:** news, war, social networks, network analysis, content analysis, mediatization

## Abstract

This contribution, focused on narratives of the Russian-Ukrainian conflict, was carried out on an annual basis of articles published in two of the main Italian newspapers (in the online version), and of comments extracted from Twitter/X. The work looks at communication structure, language and content, and at possible points of contact between the two sources, as different forms of communication: one unidirectional and institutional, the other potentially bidirectional and choral. To understand the representations of the war in the press, we chose to focus on news headlines, due to their ability to capture attention through their language, which is generally direct and clear, and capable of summarising the content of the article. The selection of Twitter to reconstruct users’ opinions is due, instead, to the specific role played by this platform in capturing opinions and attitudes with respect to the news disseminated by the selected newspapers. Useful information is extracted, with particular attention to the contents (it is thus preferred to reduce the number of comments by avoiding sentiment analysis in favour of a more controlled automatic content analysis procedure). The results show greater heterogeneity of sources and thematization on Twitter/X.

## 1 Introduction

When Russia invaded Ukraine (24 February 2022), the world was still reeling from the COVID-19 pandemic. The return to normality was interrupted by news of the war, which became a priority for the media. Many newspapers created *ad hoc* sections on the “Russia-Ukraine War” (Wall Street Journal, Washington Post, La Repubblica…), with comprehensive coverage of the conflict.

War, in general, also impacts public communication processes and produces an increasing demand for information, even in countries not directly involved. The invasion of Ukraine has generated worldwide attention (Maurer et al., 2023; Udris et al., 2022 in Schumacher et al., 2024), and it is not surprising that the conflict has been a prominent topic in international media coverage since 2014 (Fengler et al., 2020), while ongoing developments are also widely discussed on social media, which play an important role in war and conflict communication (Stieglitz et al., 2015).

Just to name a few examples, The New York Times gained 1,000 new subscribers per day in the first 2 weeks of the war, while BBC News’ live page on Ukraine recorded almost 400 million views between February 24 and March 13 (Pavlik, 2022). There was extensive coverage of the war in the Italian media as well. For example, Rai news programs devoted 70% of their coverage to the conflict, with a predominantly political, military and humanitarian focus (Milazzo, 2022).

Moreover, the public’s interest in news increased considerably, especially in the first weeks of the conflict. Many people got their information through social media, which is increasingly used as a means of information, especially by younger people, leading to greater exposure to misinformation (Gili and Giovanni, 2018).

The Russian-Ukrainian conflict has been defined as a hybrid conflict (Snegovaya, 2015; Vihalemm and Juzefovičs, 2023), because if the basis of traditional battle remains the same in the 21st century, many of the principal components of war have a digital foundation.

This war has been defined as the first commercial space war, the first full-scale drone war, and the first AI war. As an example of this last point, intelligence on Russian troops’ border movements and military plans was leaked on social media immediately before the invasion. Ukrainian civilians also use social media and special apps to report Russian activity to their army. Finally, videos recorded on smartphones with location and time data are shared online and can even be used as evidence in international courts (Karalis, 2024).

Digital networked communication enables the immediate tracking of developments and commentary on the war. Twitter, in particular, is an important arena for wars and conflicts where emerging political issues are debated and opinions are formed (Jungherr et al., 2019). The widespread use of the platform by political-media actors impacts information flows and news production, as well as agenda-setting processes (Broersma and Graham, 2016; Kapidzic et al., 2022; Schumacher et al., 2024) and can shape public perceptions and interpretations of wars (Meißner, 2019; Schumacher et al., 2024).

As a key segment of the *hybrid media system* (Chadwick, 2017), Twitter is considered a “borderless news environment” (Ojala et al., 2016, p. 2). Journalists worldwide actively use it to report on the war, making the platform a key player in shaping the perception of the war (Fahmy et al., 2022). Political journalists play a central role in disseminating models of interpretation, and are among the most visible actors on Twitter (Mourão, 2015). In hybrid warfare, not only physical attacks but also episodes of information manipulation are important tools in the struggle for power. According to some scholars, information warfare is central to hybrid warfare (Snegovaya, 2015). Hybrid warfare is not only about media coverage of the actual conflict situation, but any damage done to the enemy through media communication.

At the same time, the amount of fake news has generated confusion about the events and possible consequences of the war, also considering the widespread tendency of the media to leverage the emotional dimension more than the rational (Censis, 2025). Both covid-19 and the war have confirmed the ability of fake news to influence public opinion even when it is denied (Martellozzo, 2022).

This paper refers to the thesis of the “mediatization of war” to highlight the active and performative involvement and constitutive role of the media in contemporary conflict. This perspective goes beyond viewing the media as neutral transmitters of information; instead, it sees them as actors that help shape the visibility, emotional resonance, and perceived legitimacy of war narratives. Media do not simply reflect conflict, they participate in its construction, by selecting, framing, and amplifying certain events, voices, and symbols over others.

As Cottle (2006) notes, the media are deeply implicated in conflicts because they disseminate not only images but also ideologically charged interpretations of war. Collective interests and cultural identities that compete for political legitimacy and social recognition increasingly do so through both traditional and digital media. In this sense, media platforms become symbolic battlegrounds, where narratives are contested, identities are performed, and publics are mobilized. As Cottle (2006) observes, everyone from elected officials to activists turns to the media to pursue strategic goals and symbolic visibility.

Recent developments in digital communication, particularly social media, have further intensified this performative dimension. For instance, the study by Chen and Ferrara (2022) tracking the Twitter discourse around the Russia–Ukraine war illustrates how online platforms serve as real-time arenas for discursive conflict, where competing narratives proliferate, emotional responses are curated, and the boundaries between civilian witness, propaganda, and information warfare blur. These dynamics exemplify how media can actively co-produce the social realities of war, shaping public sentiment, political alignments, and even perceptions of truth.

Starting from these considerations, the proposed work aims to make an original contribution to the ongoing debate on the role of content transmission and its reinterpretation through social media, particularly by focusing on the potentially significant role of the power underlying information control. The effects to be analyzed and the related considerations can be multiple. Here, we focus on the analysis of the control of information disseminated by traditional media (editorial control) and social media (algorithmic or user-centered control).

The work describes these dynamics through the analysis of communication on the Russian-Ukrainian war, comparing the narratives emerging in two of the main Italian newspapers, *Corriere della Sera* e *La Repubblica*, with those found on the social network X (formerly Twitter). The attempt is to identify mechanisms through which old and new media actively shape war narratives, visibility and public sentiment.

The selection of the social network is due to the specific characteristics of forms of communication, typical of users with a desire for political debate, interest in politics, and/or active involvement, not excluding the possible presence of messages of institutional origin (Leifeld, 2018).

It emerges that platformization allows for broad participation, democratizing communication, but introducing new challenges such as misinformation, polarization, and the spread of harmful content. This shift raises questions about processes of regulation, accountability, and oversight, as platforms wield significant power in shaping public discourse, raising concerns about censorship, bias, and the need to identify transparent governance mechanisms. Various studies describe the risks linked to the management of these processes by platforms often operating on commercial models which give priority to individual interest and advertising revenues, promoting the dissemination of polemical, clearly distorted or unreliable, and poorly diversified content. Traditional media are pressured to adapt to these new distribution models and compete within the platform-driven ecosystem. However, they remain an essential tool and, as will be seen, a greater guarantee of informative content. In summary, the platformization of the public sphere, together with the platformization of news (van Dijck et al., 2018), represents a significant shift in the way public communication is structured, mediated, and experienced, with profound implications for democracy, public opinion, and social interaction.

The acceleration of digitalization processes and the institutionalization of digital platforms have profoundly changed the public sphere, long dominated by traditional media and journalism. The advent of social media has offered new spaces for public discussion on many topics. However, the initial idea that platforms would allow everyone to take part in the public sphere soon proved to be groundless, also considering the search for economic business models by platforms, which hinders real inclusion and equal participation (Fischer and Jarren, 2023).

Information provided by traditional media can be disseminated, shared with others, commented on and evaluated on social media. Furthermore, there are numerous partial publics or small user communities on the platforms which have a significant impact on the visibility of actors and topics, on the progress of discussions and on the formation of opinions. Due to the sheer volume of information provided on different channels, minorities may still have difficulty reaching broader audiences (Fischer and Jarren, 2023).

Given these premises, the advent of platforms has changed the public sphere, which is becoming increasingly diversified in terms of space and time, as well as more dynamic: more people gain access and more topics are discussed. Online platforms are also changing the structure of communication in the public sphere with implications for actors, media products, and contents, with consequences for social mediation and democracy (Fischer and Jarren, 2023).

These processes have also produced a transformation of the norms and rules of journalism, encouraging media to adapt to the many new distribution channels. Quality media outlets seek to expand their reach with attention-grabbing posts on social media. News is delivered in snippets 24/7: posts instead of articles, interviews, or features.

Just as mass media once forced politics to adapt to the mode of media production, platforms are now triggering a change in journalistic rules. Traditional media have become “platform complements” (Fischer and Jarren, 2023).

As long as the mass media dominated the public sphere, journalism contributed significantly to a controlled and trusted intermediary system. Journalists have always selected, adapted, and produced content for different distribution channels. However, social media platforms, over which journalists have less and less control, have evolved “beyond their role as distribution channels” (Bell and Owen, 2017). They undermine the gatekeeping function of journalism and transform publishers’ relationship with the public. This epistemological shift has been called the platformization of news (van Dijck et al., 2018). It follows that today news organizations select, adapt, and produce news in line with the logic of the platforms. When disseminating news on Facebook, for example, news media select “soft” articles or adapt news language to increase engagement. Social media platforms have, in fact, become central to how the public accesses news. This development has not only changed consumption habits and audience experiences but also journalistic routines: news outlets increasingly use Facebook, Instagram, TikTok or Twitter to spread news and reach new audiences (Hase et al., 2022). This process is not without negative effects, such as hate speech and forms of polarization in public communication (Bessi, 2016; Fischer and Jarren, 2023; Ross Arguedas et al., 2022).

## 2 Materials and methods

The paper compares narratives from the two main Italian online newspapers (il Corriere della Sera, la Repubblica) with those emerging from communication on social X. To compare communication registers and understand related dynamics, uniformity of language is required, so we selected only Italian comments before proceeding with the content analysis.

Two hypotheses are proposed for testing here, concerning mainly the control of information flows and differences between the structure of communication on online newspapers and social media:

- H1: First, concerning the goal to assess whether the language of war news and its main patterns change according to the type of communication, *the hypothesis is that newspaper communication exhibits greater uniformity and a specific language*. This also depends on control over sources and so this hypothesis is that editorial control in traditional media results in more coherent and verified narratives. While traditional media have seen the emergence of control tools in various countries, there is a lack of verification of information on the Internet. We want to understand how narratives about the war emerge and differ, in newspaper news and also in comments on X (Chen and Ferrara, 2022). If in the former case, information is unidirectional and controlled, in the latter, the user himself can construct or even distort it (Bessi and Quattrociocchi, 2015; Crouch, 2020).
- H2: Then, we want to assess the weight of ‘confirmation bias’ as a function of using social networks to seek information. ‘Confirmation bias’, in fact, is a reinforcement of false beliefs and prejudices due to the selection of information through an algorithm, showing messages with the same contents for each profile of users (Bessi and Quattrociocchi, 2015; Del Vicario et al., 2016). *We hypothesize that the effect of ‘confirmation bias’ increases when the perception of personal risk increases (economic crisis, political instability, migration crisis, etc.).* In particular, we want to explore how algorithmic and user-driven control in digital platforms contributes to fragmented, emotionally charged, or polarizing content—especially when risk perception is high. Studies have shown that this problem emerges with the use of social networks to the extent that the selection of information displayed by the user depends on the action of an algorithm reproducing content already viewed or in which the user has shown interest (Kiri Gunn, 2021; Ross Arguedas et al., 2022). While these dynamics do not necessarily produce echo chambers or filter bubbles, the polarized or unaware users will be subject to overexposure of information that could condition their behaviour, opinions and beliefs. This happens especially when social networks become an all-encompassing experience and the main form of information acquisition. According to some scholars, this kind of user prefers to select information with high critical or polemical content even if the source is unreliable (Del Vicario et al., 2016; Rhodes, 2021). We want to find out if hate speech and polemical comments increase when the perception of risk increases, also considering the impact of narratives in newspapers.

The collection of information and comments begins with the Russian invasion of Ukraine on February 24, 2022 and ends one year later. To allow a comparison of the narratives, both newspaper articles and social media information (original posts, replies, quote tweets, etc.) were extracted. The extracted articles contain the words ‘Russia’ and ‘Ukraine’, while the social media content is extracted through the hashtags ‘war, Russia, Ukraine’.^1^

The first phase precedes that of content analysis, and different procedures are used for the different sources. The extraction of news in newspapers involved manual selection procedures, different from those used for social media. It is indeed useful to show the numerical weight of the themes and reference lemmas. We then proceed to a preliminary descriptive analysis of the extracted news. We focused on the weight of war news detected in both newspapers, on the evolution of the debate over the time considered, and on the editorial choices relating to the thematic sections.

Regarding the communication extracted from social media, the first phase required an automatic extraction of posts and related information. The number of comments and languages used makes it impossible to proceed with a merit assessment. We therefore proceeded with an automated extraction by keyword and then used Network Analysis tools for identifying the weight of the posts, the main referents, the structure of the communication plan, and the incidence of self-referentiality. The subsequent content analysis proceeds in parallel and with a comparative purpose, identifying two corpora: the news in the newspapers and the Tweets on the social network.

### 2.1 News in newspapers

This part of the analysis refers to online news that, in recent years, has witnessed a growth in interest from the population, amplified by the pandemic, in parallel with the further decrease in readers of paper newspapers (Istat, 2022; Newman et al., 2022).

To understand the representations of war, we chose to focus on news titles, which have been the subject of numerous studies (among others Van Dijk, 1988, 2008; Reis et al., 2015), by their ability to capture attention through language which is generally direct and clear, capable of summarizing the content of the article. According to Van Dijk, important characteristics are the position of the title, the vocabulary used, and the number of occurrences of the same event.

Since they are the first element with which the reader comes into contact, titles reflect the agenda and discourse of the newspaper. Furthermore, it is worth noting that the amount of attention dedicated to online content is decreasing drastically, leading many users to read quickly (Reis et al., 2015) often focusing only on the headlines and subtitles of the news. Furthermore, in response to this trend, newspapers have over time adopted the formula of the short headline, which is launched like a slogan (Amodeo, 2002). This makes it particularly important to investigate the language and the prevalent terms chosen by newspapers to tell the story of the Russian-Ukrainian conflict, towards which there are high levels of attention.

All news items from the chosen period containing both the words “Russia” and “Ukraine” were selected using the advanced search tool of the newspaper engines.^2^ After the verification and cleaning phase, the search engines of the two newspapers returned 517 articles for Repubblica (of which 330 from February 2022 to September 30, 2022 and 186 from October 1, 2022 to February 24, 2023) and for Corriere 880 articles (of which 333 from February 24, 2022 to September 30, 2022 and 529 from October 1, 2022 to February 24, 2023), for a total of 1.397 articles.

The sections to which the articles refer are mainly foreign (201), politics and economics (100) for La Repubblica; front page (249), national (224), foreign (139) for Corriere della Sera, demonstrating not only the high importance attributed to the war and the need to highlight it in the most visible part of the website, but also to emphasize the possible repercussions of the conflict on a national level.

From the selected titles, a corpus was created and analyzed using T-Lab, a content analysis and text mining tool,^3^ with which it was possible to perform a series of processes both on the entire corpus^4^ and on the subsets with the titles of the two newspapers. The corpus was divided into quarters, with the following distribution of titles (Table 1):

**Table 1:** Number of titles by newspaper and quarter.

Quarter	Start date	End date	N. Titles Repubblica	N. Titles Corriere	Total
1°	Feb 24–2022	24-mag-22	253	238	491
2°	May 25 2022	24-ago-22	58	85	143
3°	August 25 ago2022	24-nov-22	107	257	364
4°	November 25 2022	24-feb-23	99	300	399
Total			517	880	1,397

**Figure 1:**
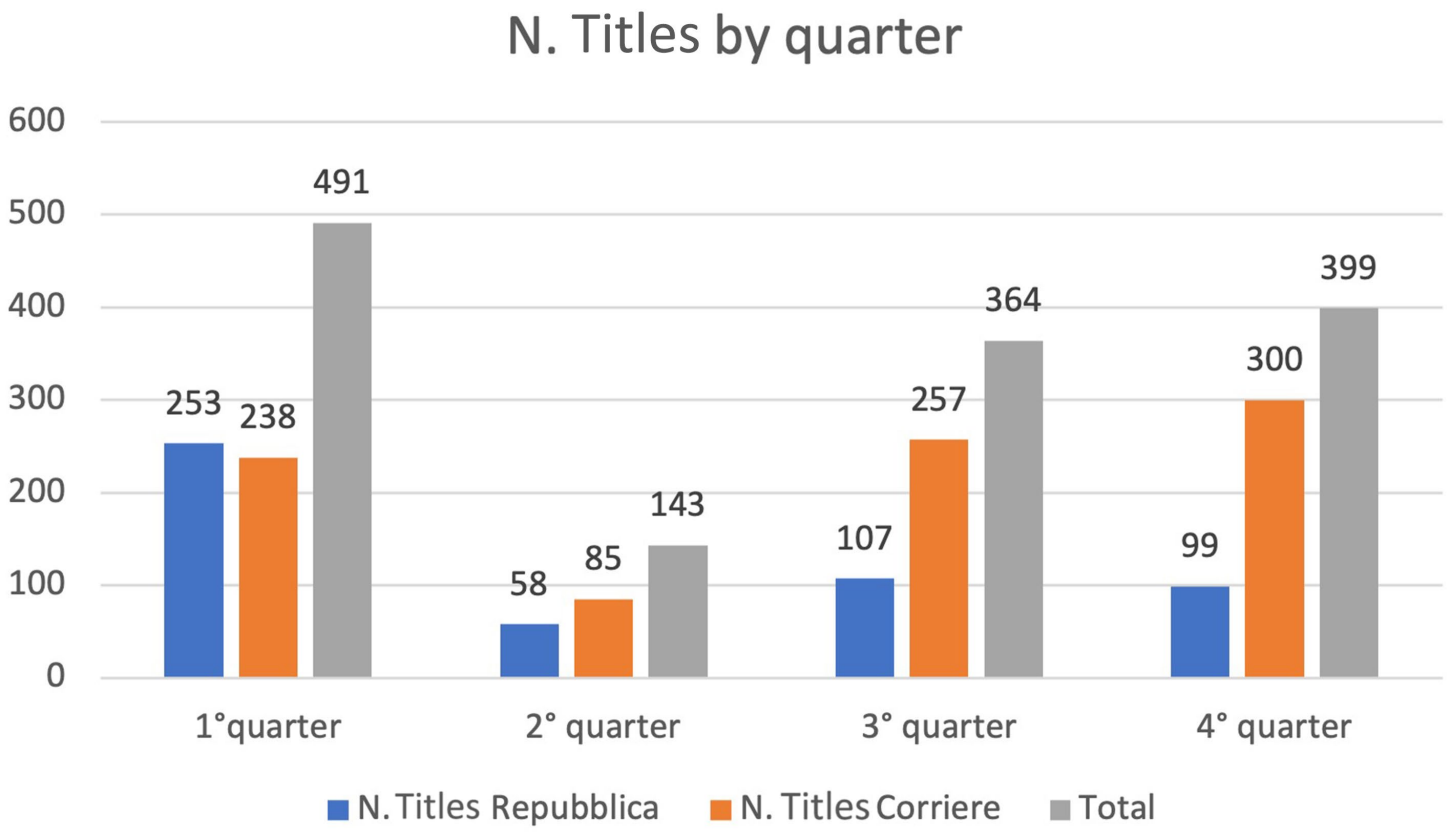
Number of newspaper titles per quarter.

As can also be seen from the Figure 1, the news of both newspapers is concentrated mainly in the first and, a long way behind, in the fourth quarters, with a prevalence of Corriere’s titles, which has dedicated a greater overall amount of news to the war.

The first quarter contains all the articles on the recent invasion, the territories affected and the consequences of the war and the fourth includes titles that discuss new attacks, the risk of the alarm of atomic war and the difficult path of negotiations (Table 2).

**Table 2:** Network analysis tools: relations among users.

NA mesaures	Feb 24^th^ -May 2022	June-Aug 2022	Sept-Nov 2022	Dec-Febr 2023
Coeff. of clustering (average)	0,1	0,1	0,1	0,1
N. Connected Components	10,853	10,966	10,962	8,765
Single Connected Components	4,902	4,467	4,344	3,720
N max of users for Components	36,211	36,634	38,927	34,011
N max of ties for Components	102,563	99,081	99,988	106,056
Centrality Betweenneess (average)	57,955	520,924	57,765	42,928
Diameter (max dist among users)	29,33	29,67	29,33	24,67
Min. dist among users (average)	9,05	8,45	8,69	7,68

The reduced attention of newspapers after the first quarter confirms the news cycles model, that predicts a quick loss of media attention for a topic after an initial or repeated peak of coverage (Fengler et al., 2020).

### 2.2 Social media communication

A source that is becoming increasingly relevant for capturing the widespread climate of opinion is that of social media communication. In this section, we try to detect differences between the contents and structures of communication on social media and newspapers (H1). The social network is more like a large virtual “square” where users from different social backgrounds, education, orientations, skills, interests, etc., meet each other. The users of social media are thus only partly attributable to the category of an engaged audience with medium to high levels of education, typically interested in newspapers. Social media content is also not often the expression of columnists and journalists, although in some cases it may be. Furthermore, digital communication, consistent with the metaphor of the public square, has a relational structure, from which it arises and with which it evolves. There is, moreover, no boundary related to language or nation, which we will impose later with the content analysis, maintaining a parallel with the analysis of Italian newspapers. We note that, in the case of social networks, it is not possible to speak of ‘public’ by attributing the same meaning to the term as we do for newspapers: here, information tends to be self-produced, and it is difficult and sometimes impossible to separate sender and receiver. At the same time, information, as is well known, is not always reliable, and even information derived from official sources can be reconstructed to the point of distorting its meaning (Corner, 2017; Lewandowsky et al., 2017).

We first show the volume of posts and users referring to the Russian-Ukrainian war. For comparative purposes with the newspapers, we proceed with an analysis by quarter. The extraction of comments begins with the outbreak of the war. The extraction of samples consists of a maximum of 32,000 users per month, a structural limit imposed by Twitter for the extraction of comments. The total number of users extracted is therefore not particularly relevant, while the structure of communication and content is more interesting.

By reconstructing the underlying relational dynamics, a certain balance emerges in the structure of communication. The number of ties allows us to detect the weight of communication between users. The total number of users in the network is, in part, composed of those who simply post material (unique links) and/or retweet it (self-loops), without receiving comments. This behaviour does not produce actual *communication* as much as what we might call *network information* dissemination, in a broad sense, or even self-referential reactions, in the case of self-loops. The presence of ties with duplicates, which considers comments to posts, views, likes, etc., allows us to discriminate, in the sense indicated above, between ‘communication’ (ties with duplicates, increasing during the first months) and ‘information’ (single ties or self-loops, almost stable).

**Figure 2:**
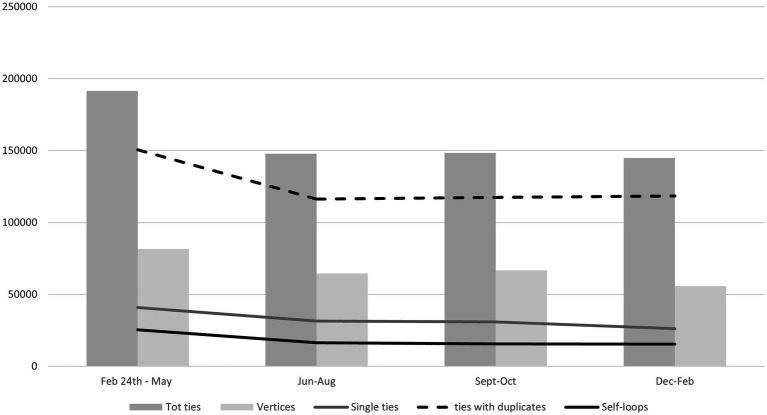
Structure of ties on Twitter/X.

In general, the presence of single (i.e., unidirectional) ties makes up about one-fifth of the recorded ties and does not show significant fluctuations (Figure 2). This means that in general, only a very small part (one-fifth) of the detected ties takes on the structure of a unidirectional information flow. The propensity to reciprocate the link, on the other hand, shows the presence of actual communication and thematic comparison. It characterizes about four-fifths of the detected flow, especially during the first part of the extraction, with the explosion of the conflict between February and May 2022. What prevails, then, is the presence of a communication structure that remains fairly homogeneous, with limited presence of self-referentiality (*self-loops*) and a high presence of confrontation, in terms of reciprocity (ties with duplicates). This is not an obvious outcome and indicates the persistence of great interest and a need to share or discuss this topic.

However, regarding the lesser or greater propensity to structure parallel communities of users into connected sub-graphs (groups), the *clustering* coefficient^5^ does not show a particularly high value and it remains homogeneous over time, showing that the propensity to interact-a fairly widespread tendency-does not lead to the formation of internally homogeneous communities (Table 3).

**Table 3:** Lemma specificity—La Repubblica.

Lemma	SUB	TOT	CHI2	(p)
Food	6	4	9,298,648	0,002
Mariupol	13	4	8,244,269	0,004
Mediator	4	5	6,195,432	0,012
Income	4	9	6,195,432	0,012
Sorry	4	9	6,195,432	0,012
Taiwan	4	14	6,195,432	0,012
Square	5	11	4,895,694	0,026
Save	5	121	4,895,694	0,026
Salvini	5	121	4,895,694	0,026
Negotiations	10	4	4,745,215	0,029
Stocks	3	4	4,6,452	0,031
Down	3	4	4,6,452	0,031
Women	3	7	4,6,452	0,031
Entrepreneurs	3	38	4,6,452	0,031
United	3	13	4,6,452	0,031
Russian	14	4	4,534,108	0,033
Invasion	7	4	3,973,937	0,046

Furthermore, the connection between components decreases in the second and fourth quarters, but in the second and third quarters the maximum number of users and links per component increases. These values will decrease in the last monitored quarter, indicating a reduction in online communication. The second quarter is also distinguished from the third by a lower weight of the centrality of intermediation (the ability of some users to mediate, becoming points of reference) and the distance between users. This structure characterizes rather closed and self-referential forms of communication. In fact, as will also emerge from the analysis of the content, in this period communication on social media takes on its own specificity: the presence of conspiracy references, distorted or completely unlikely news, absurd connections (for example, war and COVID), threats, and colloquial or explicitly offensive language increases. This happens in a phase in which, even in the newspapers, fear for the living and economic conditions of Italians emerges, and threats of Russian retaliation become explicit, fueling fears about the effects that the war could have on the economy and life in the Italian context. To observe the weight of homophilous dynamics, we finally try to identify groups with respect to the more or less similar structure of relationships, applying the Clauset-Newmann algorithm through the Nodexl application. This analysis was carried out for each month and confirms that the self-referentiality underlying communication is limited. Groups consisting of users who simply retweet content (self-loops) are, in fact, limited overall. However, the group of self-referential users constitutes the main component of the network structure in the last quarter analyzed. In this period, therefore, communication begins to break down to make room for the greater presence of self-referentiality and redundancy of information in the network. The fragmentation into small discussion groups is particularly evident in June, marking the beginning of the quarter characterized by fears and distorted communication.

## 3 Results

The analysis of news headlines in newspapers started with the reconstruction of the dictionary (disambiguation, synonym homologation, correction, etc.) of the corpus and the selection of the keywords on which to carry out further processing. The lemma^6^ that occurs most frequently is ‘war’, with 179 occurrences, compared to the 55 occurrences in which ‘peace’ occurs, followed immediately by ‘Putin’ with 177 occurrences, compared to the 39 occurrences in which ‘Zelensky’ occurs. The high presence of the term war is also linked to the decision of the Italian press to immediately recognize and denounce what was happening and characterize it as such, in the face of the denialist^7^ and repressive attitude of the Russian authorities (Lami, 2022).

‘Ukraine’ and ‘Russia’ appear 150 and 116 times respectively, ‘Moscow’ with 104 occurrences and Kiev with 83. ‘Europe’ has 81 occurrences, while ‘Italy’ occurs 58 times. The binomial ‘Russia-Ukraine’ appears 33 times and refers exclusively to Repubblica. The term ‘crisis’ is also present on average with a significant number of occurrences (34 occurrences) and the topic of energy (the term ‘gas’ 34 times and ‘energy’ 28 times).

It was possible to analyse co-occurrences^8^ for the most frequently or most significantly occurring lemmas.

**Figure 3:**
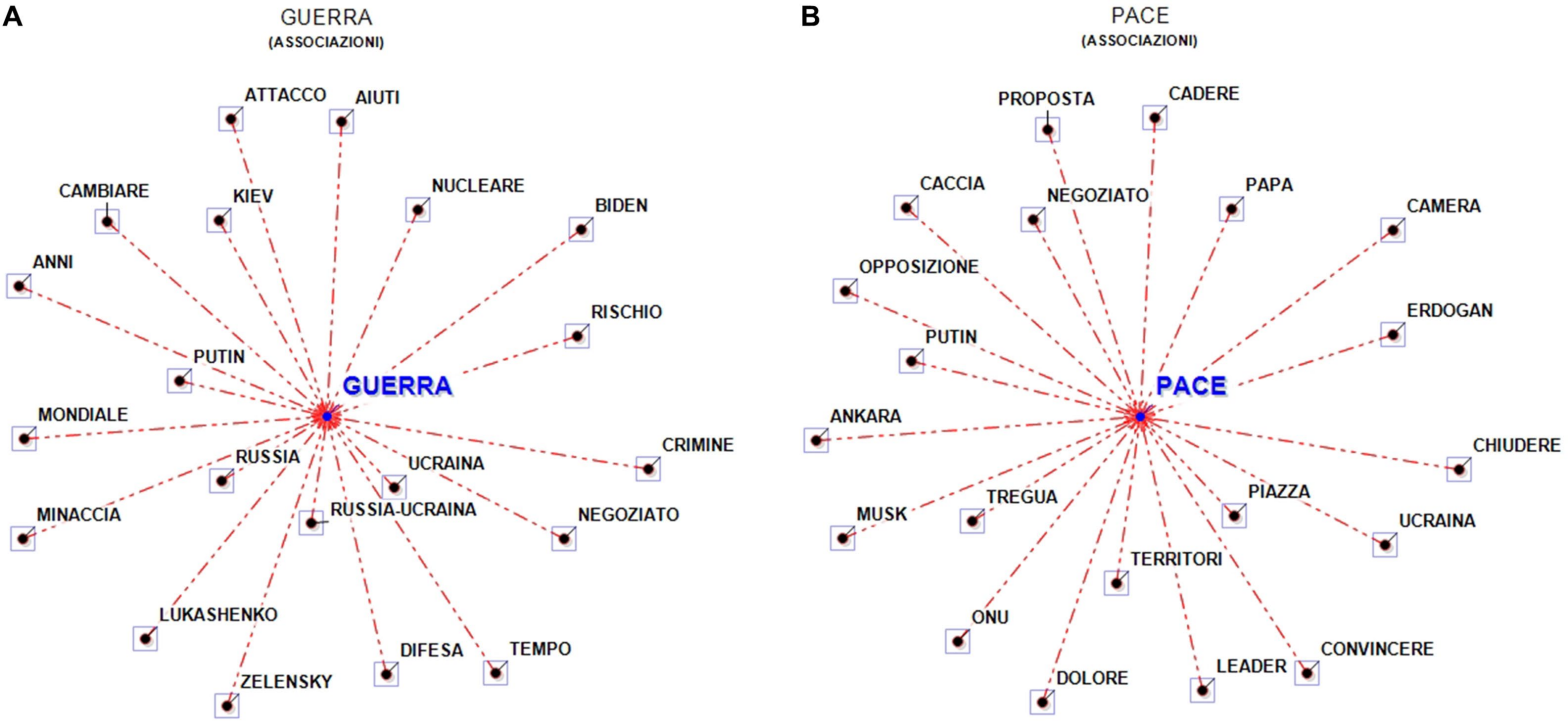
Word associations—**(A)** war and **(B)** peace (overall corpus). In **(A)**, the Italian words Attacco, Aiuti, Nucleare, Rischio, Crimine, Negoziato, Tempo, Difesa, Minaccia, Mondiale, Anni, Cambiare can be translated as follows: Attack, Aid, Nuclear, Risk, Crime, Negotiation, Time, Defense, Threat, World, Years, Change. In **(B)**, the Italian words Proposta, Cadere, Papa, Camera, Chiudere, Piazza, Convincere, Leader, Territori, Dolore, Tregua, Opposizione, Caccia, Negoziato can be translated as follows: Proposal, Fall, Pope, Chamber, Close, Square, Convince, Leader, Territories, Pain, Truce, Opposition, Hunt, Negotiation.

The lemma war is clearly associated with Russia and Ukraine. Words close to the main lemma are also Putin, risk and nuclear. Further away are negotiation, defence, aid. The lemma peace is associated with square, territories, truce, negotiation, pope, UN (Figures 3A,B).

**Figure 4:**
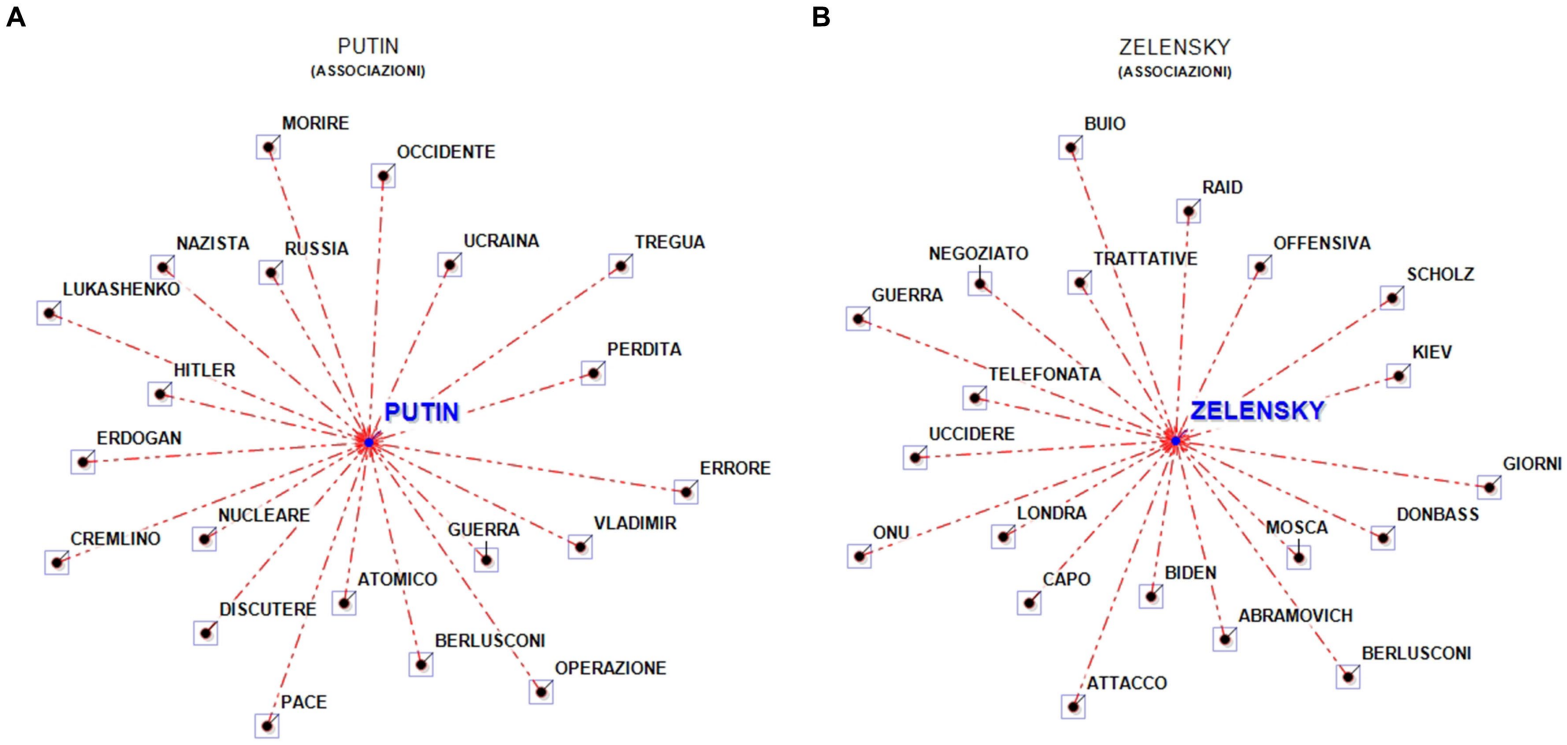
Word associations—**(A)** Putin and **(B)** Zelensky (overall corpus). In **(A)**, the Italian words: Morire, Occidente, Tregua, Perdita, errore, Guerra, Operazione, Atomico, Pace, Discutere, Nucleare, Cremlino, Nazista, Russia can be translated as follows: Die, West, Truce, Loss, Mistake, War, Operation, Atomic, Peace, Discuss, Nuclear, Kremlin, Nazi, Russia. In **(B)**, the Italian words Buio, Raid, Offensiva, Giorni, Attacco, Capo, Uccidere, Telefonata, Guerra, Negoziato, Trattative can be translated as follows: Dark, Raid, Offensive, Days, Attack, Boss, Kill, Phone Call, War, Negotiation, Negotiations.

The lemma Putin is associated with atomic, nuclear, Hitler, Nazi. Further away are the words West, peace and truce, negotiation. Putin is most associated with negotiation, resistance (Figures 4A,B).

Through the analysis of specificities^9^ and the selection of the quarter variable, we can trace the evolution of the language over a year. In particular, the terms ‘war’ and ‘Ukraine’ characterize the first quarter. The following quarter is characterized by references to the economic repercussions. In the third quarter, terms linked on the one hand to the invaded territories and the involvement of civilians, on the other to the role of the United States, but also to concerns about an escalation of the conflict prevail. Finally, in the last quarter, in addition to the theme of aid and truce, the European plan and the national political-governmental dimension dominated. The same analysis allows us to identify the typical lemmas of each newspaper: the “Corriere” headlines are most characterized by the use of the term ‘Zar’ to indicate the Russian president, the references to ‘raids’ (essentially Russian raids on Ukrainian civilians), ‘Kherson’, ‘businesses’, with reference to the consequences of the war on the Italian economy, ‘peace’, also concerning the appeals of Pope Francis and partly to the demonstrations in the square, ‘president’, essentially referring to national and foreign policy and the issue of military aid to Ukraine (Table 4).

**Table 4:** Lemma specificity—Corriere della Sera.

Lemma	SUB	TOT	CHI2	(p)
Zar	28	4	18,25,019	0
Kherson	18	58	11,69,742	0
Scholz	9	11	5,833,116	0,015
Front	8	4	5,183,456	0,022
Businesses	11	38	4,830,586	0,027
Opera	7	8	4,534,182	0,033
President	7	4	4,534,182	0,033
Choice	7	5	4,534,182	0,033
Bombs	13	10	4,247,478	0,039
Raid	10	6	4,212,518	0,04
West	6	9	3,885,292	0,048
Heroes	6	7	3,885,292	0,048
Atomic	6	42	3,885,292	0,048

The titles of “La Repubblica” are characterized more by terms linked to war territories, to issues linked to the food crisis (the increase in the price of wheat, following the Russian naval blockade, also taken up about the decrease in family income) and to the demonstrations in Italian squares against the war and to the political positions of some right-wing exponents towards Russia (Table 3).

What has been said emerges more specifically from the reconstruction of the thematic clusters, within which a series of ‘elementary contexts’ are found, the weight of which is represented in Table 5. It was possible to identify four clusters:

**Table 5:** Cluster by newspaper.

Newspaper	Cluster				
	Negotiations	Economy/Energy	Escalation	Battlefront	Total
Repubblica	19,55%	18,41%	39,77%	22,27%	100%
Corriere	24,84%	23,91%	27,34%	23,91%	100%
Total	22,69%	21,67%	32,41%	23,24%	100%

The first cluster, defined as ‘negotiations’, includes the titles of the articles that discuss the position of the governments of Moscow and Kiev and the possible negotiations in favour of the end of the war, the role of Italy and other countries in supporting Ukraine regarding the shipment of weapons.

The second cluster, defined as ‘energy’, groups together the titles of articles that focus on the economic consequences of the conflict: the increase in food prices with particular regard to the wheat issue, inflation, and the consequences on families and businesses, due to the increase in the cost of energy. The third cluster, defined as ‘escalation’, concerns fears related to the extension of the conflict on a global scale, Putin’s use of atomic bombs, and the involvement of Italy and Europe. The cluster also includes the titles of articles discussing European and US sanctions on Russia.

The last cluster, defined ‘front’, includes titles that discuss the invasion, the outcomes of the war, with reference to the number of soldiers and civilians killed, the wounded in Ukrainian hospitals and the territories of conflict.

### 3.1 The connected public and war

The last phase of the analysis concerns the content of the Tweets on X/Twitter and aims to identify the main narratives of public opinion and any changes over time. To this end, only Tweets in Italian were selected. In this sense, we will again propose an analysis that should be read in comparison to the analogous one referring to the content of the news in newspapers. In the case of social media, an analysis of the specificities is not necessary. Instead, we will try to discriminate the content characterizing each quarter.

The keywords identified for each extraction range between 2,500 and 2,800. Each keyword characterizes, in turn, a large section of lemmas and phrases with which it is associated (Table 9). This has allowed us to identify the main co-occurrences and related elementary contexts, grouped into thematic clusters. The distinction by quarters allows us to highlight how the weight attributed to specific issues and the impact of news on public opinion changes (Table 6).

**Table 6:** Lemmas characterizing communication on Twitter.

Feb–May 2022		June–Aug 2022		Sept–Nov 2022		Dec 22–Feb 2023	
Item (*n* = 2,664)	*n* occ	Item (*n* = 2,327)	*n* occ	Item (*n* = 2,816)	*n* occ	Item (*n* = 2,706)	n occ
Democrazia	19,845	Putin	11,487	Putin	12,606	Putin	14,133
Putin	16,714	Russo	11,272	NATO	7,093	NATO	9,386
Zelensky	10,672	Armi	8,487	Zelensky	6,994	armi	7,847
Usare	8,164	Italiano	7,945	armi	6,972	Zelensky	7,557
Armi	7,630	NATO	7,678	russi	6,380	Italia	6,006
NATO	5,983	Sanzioni	6,610	Donbass	4,227	russi	5,490
Pace	5,380	invasione	6,408	sanzioni	4,035	Europa	4,927
Paese	4,983	Ucraino	6,195	pace	3,583	conflitto	4,375
Mariupol	4,766	Europa	4,789	persone	3,540	UE	4,214
russi/o	8,555	Zelensky	4,616	Berlusconi	3,432	pace	3,976
Kiev	4,031	Grano	4,370	nucleare	3,404	Mosca	3,839

The high presence of the lemmas Ukraine, Russia and war depends on the decision to use these hashtags as an extraction key. Excluding these lemmas, some specificities that characterize each quarter stand out (Table 7):

- in the first quarter, the reference to ‘democracy’ and ‘peace’ stands out;
- in the second quarter, the reference to sanctions, military action and the Italian context takes on particular significance. This is a period in which the fear that war could affect the well-being and standard of living of each person intensifies. Fears for oneself, possible deprivation and the increase in prices increase while the word ‘peace’ does not emerge as a significant item;
- in the third quarter, both the reference to the fear of nuclear power and the call for indignation and moral condemnation for the military invasion of Ukraine emerge as relevant;
- in the fourth quarter, a certain redundancy emerges with respect to the themes already discussed.
- Otherwise, Tweets often mention the two leaders together, and therefore the co-occurrences do not sufficiently discriminate the positive and negative polarity (even if associated with the two figures). This is an important difference compared to what emerges, instead, in the analysis of the newspapers.

Further information about the reconstruction of the debate on the social network emerges through the extraction of clusters of elementary contexts (Table 7). In the second quarter, moreover, over 50% of the Tweets refer to suspicions of conspiracies or ulterior motives. It is interesting to note that, therefore, the theme of ‘peace’ is not present when the debate becomes much more focused on references to conspiracy theories, post-truth, and fears related to the possible impact of war on subjective well-being.

**Table 7:** Weight of elementary contexts (phrases) per cluster.

First quarter		Second quarter		Third quarter		Fourth quarter	
Negotiations	24.82%	Afterthoughts	35.04%	Weapons	47.14%	Russ/Ukrainians	20.01%
Invasion	16.23%	Putin	24.1%	Speeches	17.52%	Sanctions	29.15%
Sanctions	26.36%	War	17.44%	Comments	35.34%	Political Opinions	14.64%
Protests	4.63%	Conspiracies	23.42%			Social Opinions	15.47%
Media	27.97%					Italy	20.74%
Tot phrases (*n*)	108,928	Tot phrases (*n*)	94,433	Tot phrases (*n*)	95,413	Tot phrases (*n*)	99,332

The main clusters that emerged in the first quarter are: ‘Negotiations’, ‘Sanctions’, and ‘Media’. Tweets that characterise the ‘negotiations’ cluster recall indignation over bloody Russian military actions. The ‘sanctions’ cluster instead refers to assessments and fears about the possible outcomes of sanctions against Russia and related repercussions. Finally, the cluster referring to the media recalls political comments and debates by proposing or re-proposing interpretations spread by the media.

As for the less relevant clusters for the first quarter, ‘invasion’ presents polarized Tweets that refer to news reported by newspapers, while the ‘protests’ cluster groups comments against the political choices of the main protagonists of the war.

If aggressive, polemical comments, focused on conspiracy theories and distorted information, are not particularly widespread in the first quarter, they become the main ones in the second quarter. It is worth dwelling on this quarter because now communication strongly takes on the connotations of conspiracy theories, calls for ‘post-truth’ or hate speech (in particular in the ‘afterthoughts’ and ‘conspiracies’ clusters, but also the others present some comments with these characteristics). This occurs in a phase in which even newspapers, and in general all the mass media, underline the risks of individual and national repercussions, and the threats of Russian retaliation to the sanctions of Europe have great resonance in this sense. The effect on social communication seems to be the spread of conspiracy or aggressive communication (this seems to confirm hypothesis H2).

The main clusters, called ‘Backthoughts’ and ‘Plots’, are two polarities of the same thematic plane (first factor, explaining 46% of variability). The other clusters, decidedly less relevant, characterise the second factor (27.95%) of factorial analysis.

The first cluster, called ‘after-thoughts’, is the most consistent in terms of numbers and is almost entirely centered on comments to a single post, about the and questionable reinterpretations of previous mysterious Russian invasions, dating back to 2014. The ‘conspiracies’ cluster, on the other hand, has different shapes of meaning, but the reference to post-truth and conspiracy theories is widespread. All these comments are explicitly pro-Soviet but we distinguish three categories of references:

- Some users trace the issue back to local political skirmishes or to mandatory vaccination, sometimes without any logic or using tones typical of a sort of apocalyptic indifference, connecting the topic to political issues, to mandatory vaccination, to other alleged conspiracies aimed at criticizing national politicians (in Italy a link emerges—difficult to explain rationally—with anti-vax conspiracy theories).
- Other users seem to believe that the reasons for the war are linked to the choice to sanction Russia.
- Another position is that of those who do not pose any ethical problems nor recall the issue of international relations, global risks and sovereign choices but underline their disappointment with political choices considered detrimental to their own, personal interests.

The second factor, however, is weighed down by the ‘Putin’ cluster, which takes on heated and accusatory tones but underlines the inevitability of events in favour of the Soviet position. Next to this cluster is the ‘War’ cluster, the least significant in this period, which mainly reports information on the war, related risks, assessments and actions without particular opinions.

The description of the debate continues, in the third quarter, with three thematic clusters that effectively characterize three distinct groupings of themes. Also, in this case it is useful to observe the position of the clusters on the factorial plane. The clusters ‘weapons’ and ‘comments’ are located, in fact, at the two ends of the first axis (explaining the 60% of variability) as they refer to specular themes: on the one hand the issues related to the risk of extension of the conflict and global problems (nuclear risk), on the other the reference to comments of users that become redundant, referring to internal issues reinterpreted in the light of the international conflict. The polarization of the debate is reduced, as is the reference to post-truth and conspiracies. On the second factor (40%), instead, is located the cluster ‘Discourses’ that refers to the speeches of and about Italian politicians (recalling an internal projection of the international political conflict).

During the fourth quarter, finally, the clusters are redundant for what has already been described and are fragmented, while the level of opinions splits. The cluster ‘political opinions’ reiterates the polarization of the international debate by personalizing it and therefore recalling the two protagonists of the international debate.

The ‘social opinions’ cluster instead refers above all to the possible social effects of political choices, from the central role of Russian energy resources for Europe to the hypothetical new acts of aggression by Russia towards other countries, after the defeat of Ukraine. The last relevant cluster, finally, refers to the Italian territorial context. It is interesting to note how this dimension, relevant for newspapers, absolutely takes a back seat when dealing with comments on social media. Finally, it is worth highlighting the ‘mediatization’ effect of war. This particularly characterizes some clusters but assumes different functions. The cluster named ‘war’ is largely constituted by references to daily news, reported to describe the conditions and without particular evaluative or critical purposes. Other clusters, instead, recall news from the media (not necessarily newspapers), but juxtaposing comments aimed at redefining or completely modifying the original content, criticizing or ironizing. This happens especially in the clusters named as ‘after-thoughts’ and ‘conspiracies’.

## 4 Discussion

The results of the analysis confirm the initial hypotheses and offer insights into how different media systems exercise control over war-related narratives through distinct mechanisms of information management. The comparison between mainstream newspapers and the social network X (formerly Twitter) reveals how traditional and digital environments configure divergent forms of communicative control, one institutional and editorial, the other decentralized, algorithmic, and user-driven. In the case of newspapers, headlines tend to reflect a framing aligned with institutional agendas and official narratives. This editorial gatekeeping reinforces dominant interpretations of the conflict, particularly through the denunciation of Russia’s denialist and repressive stance (Lami, 2022). Such content represents a form of top-down information control, where professional norms and political proximity shape the structure and content of public discourse.

By contrast, discourse on X emerges as more fragmented and polarized, characterized by the coexistence of competing narratives. In particular, pro-Russian discourse is concentrated in conspiratorial and denialist clusters, while overall language structures reveal a proximity to personal and national frames of reference. Sentiment attribution is more ambiguous, and geopolitical hierarchies, such as the Italy–Europe relationship, are inverted in comparison to traditional media. This dynamic reflects a user- and algorithm-driven mode of control, where virality, visibility, and engagement metrics influence narrative prominence, regardless of content quality or veridicity.

The second hypothesis, concerning the greater diffusion of false, aggressive, or conspiratorial information on social media, is supported by the data, especially in the second phase of observation. This confirms the risks associated with the platformization of communication, where the affordances of digital platforms both enable broad participation and facilitate the circulation of distorted or low-quality content.

Moreover, the findings highlight the presence of a mediatization effect, where traditional and digital media mutually influence each other.

Further confirmation of this reciprocity of content emerges in the second quarter analyzed, where a convergence emerges between increased perceived fears for one’s personal situation—associated by users with Italy’s role in the conflict and the resulting threats of economic sanctions from Russia—and the increase in clusters referring to aggressive posts and comments, post-truth, and conspiracy theories, with some considerations and associations between facts and events that are sometimes completely irrational. This content, usually marginal, takes center stage and increases exponentially during this period.

So, social media not only transforms news consumption and public access to information but also contributes to redefining journalistic routines and distribution logics. At the same time, newspapers continue to be an authoritative source of information and remain central in shaping the communicative environment of social media, where they are reinterpreted, amplified, or contested by users.

In this context, the concept of control over information flows emerges as a relevant interpretive axis of the study. The war narratives produced and disseminated across these different media ecosystems are shaped by contrasting regimes of control: editorial and institutional on the one hand, algorithmic and participatory on the other. These regimes not only determine which content circulates, but also how meanings are constructed, negotiated, and legitimized within the hybrid media system.

In conclusion, the mediatization of war should be understood not only as a reflection of broader media transformations, but as an active force that reshapes the structures, dynamics, and responsibilities of public communication in the digital age, with lasting consequences for democratic debate, trust in information, and civic engagement.

## Data Availability

The raw data supporting the conclusions of this article will be made available by the authors, without undue reservation.

## Footnotes

^1^The content was extracted using the Nodexl software, through an automated extraction procedure. The choice to also include the hashtag ‘war’ for Social Network’s extraction was necessary to select only the pertinent information, reducing the number of potentially misleading comments.

^2^In particular, in Repubblica the “exact phrase” option was chosen and in Corriere the “guided” search tool was used by entering the same key words. The search for the selection of titles voluntarily excluded the term “guerra” to try to understand how much the term weighed compared to other possible words.

^3^The TLab Plus 2022 software (vers. 8.1.3.2) was used; for further information see Lancia (2004), www.tlab.it

^4^Automatic lemmatization and customized keyword selection were applied.

^5^In graph theory, the *clustering* coefficient is a measure of the degree to which nodes in a graph tend to be connected to each other. Usually, real-world (or virtual, as in this case) networks tend to create strongly clustered groups characterized by a relatively high density of connections.

^6^A lemma is defined as “a set of words with the same lexical root (or lexeme) and belonging to the same grammatical category (verb, adjective, etc.)” www.t-lab.it.

^7^The denialist attitude also finds a link in the expression used to define the Ukrainian invasion as a ‘special operation’, later replaced (only last September) with the term ‘war’, although not referring to Ukraine, but to the West.

^8^The “word associations” function processes the number of co-occurrences of words within the same sentences (in our analysis, individual titles). Through the graphic representation, the selected lemma is placed in the centre at a distance proportional to its degree of association with the other words. Therefore, the closest words indicate a greater number of times in which the chosen lemma, or keyword, occurs simultaneously with the other words. The most distant ones occur together with the keyword a lower number of times. The co-occurrences were calculated through the cosine coefficient or the association index, defined as the relationship between the quantity of co-occurrences between each pair (www.tlab.it).

^9^This T-Lab function allows us to compare the dictionary of the single corpus with the overall dictionary, to trace the typical and overused words in the single corpus. The table shows the occurrences and the chi-square value in decreasing order (www.tlab.it).
